# The ENIGMA sports injury working group:– an international collaboration to further our understanding of sport-related brain injury

**DOI:** 10.1007/s11682-020-00370-y

**Published:** 2020-07-27

**Authors:** Inga K. Koerte, Carrie Esopenko, Sidney R. Hinds, Martha E. Shenton, Elena M. Bonke, Jeffrey J. Bazarian, Kevin C. Bickart, Erin D. Bigler, Sylvain Bouix, Thomas A. Buckley, Meeryo C. Choe, Paul S. Echlin, Jessica Gill, Christopher C. Giza, Jasmeet Hayes, Cooper B. Hodges, Andrei Irimia, Paula K. Johnson, Kimbra Kenney, Harvey S. Levin, Alexander P. Lin, Hannah M. Lindsey, Michael L. Lipton, Jeffrey E. Max, Andrew R. Mayer, Timothy B. Meier, Kian Merchant-Borna, Tricia L. Merkley, Brian D. Mills, Mary R. Newsome, Tara Porfido, Jaclyn A. Stephens, Maria Carmela Tartaglia, Ashley L. Ware, Ross D. Zafonte, Michael M. Zeineh, Paul M. Thompson, David F. Tate, Emily L. Dennis, Elisabeth A. Wilde, David Baron

**Affiliations:** 1 Department of Child and Adolescent Psychiatry, Psychosomatics and Psychotherapy, Ludwig-Maximilians-Universität München, Waltherstr. 23, 80337 Munich, Germany; 2 Psychiatry Neuroimaging Laboratory, Brigham and Women’s Hospital, Harvard Medical School, Boston, MA USA; 3 Department of Rehabilitation and Movement Science, Rutgers Biomedical Health Sciences, Newark, NJ USA; 4 School of Graduate Studies, Rutgers Biomedical Health Sciences, Newark, NJ USA; 5 Department of Neurology, Uniformed Services University of the Health Sciences, Bethesda, MD USA; 6 VA Boston Healthcare System, Boston, MA USA; 7 Graduate School of Systemic Neurosciences, Ludwig-Maximilians-University, Munich, Germany; 8 Departments of Emergency Medicine & Neurology, University of Rochester School of Medicine, Rochester, NY USA; 9 UCLA Steve Tisch BrainSPORT Program, Los Angeles, CA USA; 10 Neurology and Neuropsychiatry, David Geffen School of Medicine at UCLA, Los Angeles, CA USA; 11 Department of Neurology, University of Utah School of Medicine, Salt Lake City, UT USA; 12 Department of Psychology, Brigham Young University, Provo, UT USA; 13 Neuroscience Center, Brigham Young University, Provo, UT USA; 14 Department of Kinesiology and Applied Physiology, University of Delaware, Newark, DE USA; 15 Biomechanics and Movement Science Program, University of Delaware, Newark, DE USA; 16 Department of Pediatrics, Division of Neurology, UCLA Mattel Children’s Hospital, Los Angeles, CA USA; 17 Elliott Sports Medicine Clinic, Burlington, ON Canada; 18 Department of Intramural Research, National Institutes of Health, Bethesda, MD USA; 19 Department of Neurosurgery, David Geffen School of Medicine at UCLA, Los Angeles, CA USA; 20 Psychology Department, The Ohio State University, Columbus, OH USA; 21 Chronic Brain Injury Program, The Ohio State University, Columbus, OH USA; 22 George E. Wahlen Veterans Affairs Medical Center, Salt Lake City, UT USA; 23 Leonard Davis School of Gerontology, University of Southern California, Los Angeles, CA USA; 24 Department of Biomedical Engineering, Viterbi School of Engineering, University of Southern California, Los Angeles, CA USA; 25 National Intrepid Center of Excellence, Walter Reed National Military Medical Center, Bethesda, MD USA; 26 H. Ben Taub Department of Physical Medicine and Rehabilitation, Baylor College of Medicine, Houston, TX USA; 27 Michael E. DeBakey Veterans Affairs Medical Center, Houston, TX USA; 28 Center for Clinical Spectroscopy, Brigham and Women’s Hospital, Harvard Medical School, Boston, MA USA; 29 Departments of Radiology, Psychiatry and Behavioral Sciences and The Dominick P. Purpura Department of Neuroscience, The Gruss Magnetic Resonance Research Center, Albert Einstein College of Medicine, Bronx, NY USA; 30 Department of Radiology, Montefiore Medicine, Bronx, NY USA; 31 Department of Psychiatry, University of California, San Diego, La Jolla, CA USA; 32 Department of Psychiatry, Rady Children’s Hospital, San Diego, CA USA; 33 Mind Research Network, Albuquerque, NM USA; 34 Departments of Neurology and Psychiatry, University of New Mexico School of Medicine, Albuquerque, NM USA; 35 Department of Neurosurgery, Medical College of Wisconsin, Milwaukee, WI USA; 36 Department of Radiology, Stanford University, Stanford, CA USA; 37 Department of Occupational Therapy, Colorado State University, Fort Collins, CO USA; 38 Centre for Research in Neurodegenerative Diseases, University of Toronto, Toronto, ON Canada; 39 University Health Network, Toronto, ON Canada; 40 Krembil Brain Institute, Toronto, ON Canada; 41 Department of Psychology, University of Calgary, Calgary, Alberta Canada; 42 Spaulding Rehabilitation Hospital, Harvard Medical School, Boston, MA USA; 43 Imaging Genetics Center, Stevens Neuroimaging & Informatics Institute, Keck School of Medicine of USC, Marina del Rey, Los Angeles, CA USA; 44 Departments of Neurology, Pediatrics, Psychiatry, Radiology, Engineering, and Ophthalmology, USC, Los Angeles, CA USA; 45 Western University of Health Sciences, Pomona, CA USA

**Keywords:** Concussion, ENIGMA, Repetitive head impacts, Sport-related brain injury

## Abstract

Sport-related brain injury is very common, and the potential long-term effects include a wide range of neurological and psychiatric symptoms, and potentially neurodegeneration. Around the globe, researchers are conducting neuroimaging studies on primarily homogenous samples of athletes. However, neuroimaging studies are expensive and time consuming, and thus current findings from studies of sport-related brain injury are often limited by small sample sizes. Further, current studies apply a variety of neuroimaging techniques and analysis tools which limit comparability among studies. The ENIGMA Sports Injury working group aims to provide a platform for data sharing and collaborative data analysis thereby leveraging existing data and expertise. By harmonizing data from a large number of studies from around the globe, we will work towards reproducibility of previously published findings and towards addressing important research questions with regard to diagnosis, prognosis, and efficacy of treatment for sport-related brain injury. Moreover, the ENIGMA Sports Injury working group is committed to providing recommendations for future prospective data acquisition to enhance data quality and scientific rigor.

## Sport-related brain injury

**Fig. 1:**
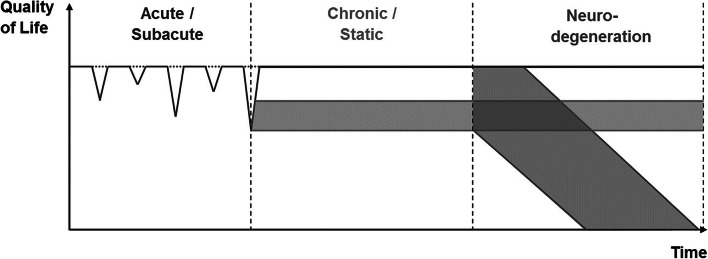
Multi-stage disease model of short- and long-term consequence following sport-related brain injury (adapted from Koerte et al. Brain Pathology, 2015). Quality of life is indicated by symptom load, which is expressed as a function of time, thereby allowing for the differentiation between at least three main trajectories of the disease including an acute/subacute phase, a chronic/static phase, and a phase of possible neurodegeneration

Sport-related brain injury is a broad term that describes alterations in brain structure and function resulting from mechanical forces to the head sustained while participating in sports. The most common forms of sport-related brain trauma are typically categorized as sport-related concussion (SRC) and exposure to repetitive head impacts (RHI) which may have cumulative effects on brain structure and function. Stages of brain alterations due to sport-related brain injury have been described as acute/subacute, chronic but static, and progressive neurodegenerative decline, as shown in Fig. 1.

Sport-related Concussion (SRC) is common in athletes. An estimated 1.6 to 3.8 million people suffer from SRC annually in the United States alone (Daneshvar et al. 2011; Laker 2015; Langlois et al. 2006). SRC occurs in all sports, but incidence rates are highest in contact and collision sports such as football, soccer, rugby, or ice hockey (Guskiewicz et al. 2000; Laker 2015; Marar et al. 2012; Meehan et al. 2010). SRC is characterized by a sudden, but typically, transient impairment in brain function following an impact to the head, face, neck, or body. Symptoms of SRC include headache, dizziness, visual ocular dysfunction, loss of memory, and confusion and resolve within weeks in adults and up to a month in children and adolescents (Iverson et al. 2017). It is estimated that about 10–15% of individuals with SRC will experience prolonged symptoms which often include headache, trouble concentrating, and sleep disturbances (McCrory et al. 2013, 2017). However, currently, there are no objective biomarkers that would assist medical diagnosis, prognosis, and recovery.

Repetitive Head Impacts (RHI) are even more common than SRC. Athletes participating in contact and collision sports are often exposed to thousands of head impacts, e.g., when heading the ball in soccer (Koerte et al. 2015b; Stewart et al. 2017). RHI may not result in acute symptoms and are therefore also sometimes called “subconcussive head impacts”. However, even in the absence of acute symptoms (as indicated by a horizontal dotted line in Fig. 1), exposure to RHI over time may have cumulative effects on brain structure and function. In fact, RHI has been associated with both transient and persistent impairment of neurological, behavioral, and cognitive function (Koerte et al. 2015b, 2015c; Montenigro et al. 2017), as well as potentially with an increased risk for developing a neurodegenerative disease (Lehman et al. 2012; Mackay et al. 2019; Nguyen et al. 2019). However, to date, little is known about the risk factors for adverse outcome and there are no known biomarkers for the prediction of clinical symptoms and dysfunction.

Taken together, given the large number of individuals participating in sports and the potential for long-lasting and deleterious symptoms of both SRC and RHI, it is important to develop biomarkers for early diagnosis, monitoring, and prognosis. A better understanding of the effects of SRC and RHI, and risk factors for long-term symptoms is needed to develop targeted treatments and improve outcomes.

In recent years, we have seen an exponential increase in research efforts aimed at understanding the effects of SRC and RHI (Bigler 2018; Hasan et al. 2018; Koerte et al. 2015b; McCrory et al. 2013, 2017; Slobounov et al. 2012). Although these studies have substantially changed how sport-related brain trauma is managed and have promoted rule changes across sports, the generalizability of findings from many of these studies is reduced due to a number of limitations. First, the majority of current studies include small samples sizes – mainly due to limited research funding – and are thus often underpowered. Second, to date, most studies focus on homogenous samples such as athletes from a single sport (e.g., American football or soccer) which may not account for anthropometric differences that could potentially contribute to the trajectory of recovery. Third, if control groups are included at all, then case-control study designs are common, where athletes with SRC or contact sport athletes (who are at an increased risk for RHI) are compared to non-athletes or athletes from non-contact sports, respectively. By doing so, potential differences in outcome measures of brain structure and function between athletes with SRC and controls are likely confounded by sport-specific factors such as differences in level of strength versus endurance training. Fourth, longitudinal and prospective studies are sparse. Most studies are limited to post SRC or post RHI-exposure measurements, thus limiting our understanding of changes occurring over time regarding symptoms as well as brain structure and function. Finally, there are only very few multi-site studies on SRC or RHI that include specific populations such as collegiate contact sports athletes only (e.g., the NCAA-DoD CARE Consortium (Broglio et al. 2017)). To date, there are also very few international multi-site projects (e.g., the ERA-NET Neuron project REPIMPACT) which would have the potential to identify generalizable effects of regional differences. While these large-scale, multi-site projects will significantly advance the field, they also require appropriate funding and often also a centralized data base which limits possible participation of collaborators around the globe.

However, it is important to improve our understanding of the clinical long-term implications of sport-related brain injury, and this field of research needs to address important questions by providing robust, reliable and reproducible data that will inform athletes, clinicians, and policy makers.

## Important questions to address in future studies

### What is the underlying physiology and pathophysiology of SRC and RHI?

Sport-related head impacts may cause shear deformation of the brain, leading to microscopic strains that may result in temporary alterations in axonal membrane permeability, ionic shifts, and impaired oxidative metabolism (Giza and Hovda 2014). Additive effects may include a decrease in total cerebral blood flow, activation of N-methyl-D-aspartate receptors, and a decrease in gamma-aminobutyric acid (GABA) and other inhibitory neurotransmitters (Filipcik et al. 2015). RHI is assumed to trigger chronic neuroinflammatory processes which, in some cases, may result in long-term neurodegeneration (Cheng et al. 2020). However, our knowledge of the underlying pathomechanisms is still limited. Identifying brain abnormalities following SRC and RHI in vivo requires the application of highly sensitive measures of brain structure and function. Such measures are extant. In fact, state-of-the-art neuroimaging techniques such as diffusion MRI imaging (dMRI), functional MRI (fMRI), positron emission tomography (PET), and MR spectroscopy (MRS), provide unprecedented sensitivity and make it possible to detect even subtle brain alterations (Koerte et al. 2015b) in vivo. As such, the inclusion of many of these neuroimaging techniques in studies of SRC and RHI have provided important insight into physiological, pathophysiological, neurological processes, and trajectory of recovery (Bigler 2018; Hasan et al. 2018; Koerte et al. 2015b; Slobounov et al. 2012). However, reproduction of findings in larger samples and across cohorts is needed and validation of imaging markers with clinical outcome is required, both of which could benefit from data or cohort aggregation.

### How can we characterize the nature of and mechanisms underlying altered brain structure and function?

Interpretation of an increase or decrease in measures of white matter organization, such as fractional anisotropy based on diffusion tensor imaging (DTI) is challenging, particularly in populations exposed to RHI, in that it is difficult to differentiate whether the changes in DTI metrics are, for example, due to neuroinflammation or to reduced neuronal integrity (Chamard et al. 2016; Henry et al. 2011; Schranz et al. 2018). Moving forward, the combination of multiple neuroimaging techniques may help to characterize better brain structural and functional changes. MRS, which allows for the measurement of metabolites sensitive to neuroinflammation (gluthathione), neuronal viability (N-acetyl aspartate), and axonal injury (choline (Alosco et al. 2019; Koerte et al. 2015a)), may further inform the interpretation of DTI metrics in the same individual. Thus, studies including multimodal neuroimaging, are likely to be more sensitive and more specific to subtle neural alterations associated with RHI and SRC. In addition, harmonization of imaging data across different cohorts will open up new opportunities to explore combination of modalities and imaging measures (Cetin Karayumak et al. 2019).

### What are the modulating factors in the development of persistent symptoms?

Recent studies have identified a number of risk factors for the development of persistent symptoms. Known risk factors include severity of acute and subacute symptoms, age at time of injury, sex, history of chronic headache or migraine prior to injury, mental health problems prior to injury, and history of previous brain injury (Iverson et al. 2017; Zemek et al. 2013). However, there are also important caveats in identifying risk factors and interpreting outcomes based on current studies. Specifically, many studies do not account for differences in pre-injury symptoms, cognitive function, behavioral health, or developmental/learning abilities, or how these multiple premorbid variables can interact to influence outcomes (Iverson et al. 2017). Still other studies lack adequate comparison groups and are limited in sample size. Future studies thus need to replicate findings on potential risk factors in larger samples and across various sports. Moreover, future studies need to investigate and identify other possible risk and modulating factors including hormonal, environmental, behavioral, and genetic factors as well as common comorbidities such as sleep issues and chronic headache. By curating and harmonizing data across cohorts, more sophisticated statistical models might be used to improve our understanding of the interaction between these variables.

### What is the influence of confounding factors such as participating in sports with differing exposure to brain trauma, training regimens, and access to medical and athletics staff?

Collision and contact sports (e.g., American football or ice hockey) are associated with higher incidence and risk of SRC than non-contact sports such as baseball or swimming (Daneshvar et al. 2011; Kerr et al. 2017). Thus, our understanding of the effects of SRC is largely drawn from data consisting primarily of male football athletes (Daneshvar et al. 2011). There are, however, considerable differences between sports including various degree of exposure to RHI and SRC, differences in training regimens, life-style, sport culture, and diet (Holway and Spriet 2011; Hootman et al. 2007). Further, there are also likely biological and genetic differences between athletes who choose to participate in different types of sports. Finally, the knowledge, training, and expertise of healthcare professionals and athletics personnel who are responsible for diagnosing SRC may further impact diagnosis and outcome of SRC as well as data quality collected in the study of SRC. This is a particular concern when collecting data in high school and recreational athletes, where only approximately 42% of high schools in the United States have access to a certified athletic trainer and others do not have access to any trained medical staff (Karlin 2011).

### What are differences in outcomes in relation to biological sex and gender?

It will be important for future studies to differentiate between variations in outcomes due to sex (biological factors such as chromosomes, sex hormones, and genetics), versus gender (behavior, lifestyle, and social and cultural norms). Female athletes may report greater frequency and severity of symptoms and are at increased risk for developing persistent symptoms (Broshek et al. 2005; Desai et al. 2019; Iverson et al. 2017). Differences in the way symptoms are being reported by males compared to females may play a role (Desai et al. 2019; Dolle et al. 2018; Iverson et al. 2017; Koerte et al. 2020). However, differences in symptoms may also be due to biological differences in brain structure and function that may lead to increased vulnerability of the female brain to shear forces (Dolle et al. 2018) or differences in the neuroinflammatory response to trauma (Villapol et al. 2017). Additionally, there is evidence to suggest that decreased neck strength in female athletes is associated with higher linear head acceleration forces when the head is impacted compared to males (Caccese et al. 2018; Tierney et al. 2005), which may increase SRC risk and severity of outcomes associated with RHI. Other work suggests that females may experience worse outcomes dependent on when in the menstrual cycle they sustained a SRC (Wunderle et al. 2014).

### What are interactions between brain development and SRC or RHI?

To date, studies investigating the effects of SRC and RHI on the developing brain are sparse. Importantly, the effects of SRC and RHI in childhood, adolescence, and young adulthood may extend well beyond the acute post-injury phase as the injury likely affects the trajectory of brain development. Even subtle chronic cognitive dysfunction may adversely affect an adolescent’s developmental trajectory and may lead to impaired psychosocial functioning and ultimately failure to obtain developmental potential (Babikian et al. 2015; Giza et al. 2005; Koerte et al. 2017). The most common long-term symptoms following SCR include emotional problems and subjective/objective cognitive dysfunction. In fact, new onset mental disorder is more frequent in adolescents with history of mTBI than in those without (Emery et al. 2016; Max et al. 2012; Sariaslan et al. 2016). Importantly, children and adolescents remain an understudied population, despite the fact that they often participate in sports and that the developing brain is particularly vulnerable to injury.

## ENIGMA approach and potential

Enhancing NeuroImaging Genetics through Meta-Analysis (ENIGMA) is an international consortium of scientists investigating the genetic underpinnings of brain structure and function in a wide range of diseases. The ENIGMA consortium currently includes nearly 1400 scientists across 43 countries around the globe investigating neurological and psychiatric diseases (for review, see (Thompson et al. 2014, 2020)). The ENIGMA Brain Injury working group was founded in 2016 and is led by Drs. Wilde, Tate, and Dennis (Wilde et al. 2019). The ENIGMA Brain Injury group includes subgroups dedicated to specific populations: pediatric moderate to severe TBI (msTBI (Dennis et al. 2019)), military-related head injury (Tate et al. 2019), intimate partner violence (Esopenko et al. 2019), adult msTBI (Olsen et al. 2019), and methods, i.e., MR spectroscopy (Bartnik-Olson et al. 2019) working groups.

The overall aim of the ENIGMA Sports Injury working group is to move the field of research forward by addressing important questions related to sport-related brain injury. In order to do so, the ENIGMA Sports Injury working group will:

1. establish a collaborative platform enabling discussion and knowledge transfer;
2. leverage existing data by enabling data sharing and analysis on the basis of transparency, rigor, reproducibility, and collaboration;
3. define key elements of the study of sport-related brain injury and provide recommendations for future research.

Below we provide more detail regarding the three aims of the ENIGMA Sports Injury working group:

1. Establish a collaborative platform: We aim to invite scientists committed to the study of sport-related brain injury from all over the world. It is our aim to serve as a platform for scientific discussion, and to actively foster collaboration between scientists with different expertise and background and at all levels of training. Our collaboration is characterized by transparency and equality. Importantly, we welcome all levels of participation depending on the scientist’s interest, background, and abilities. It is our expectation that the success of the ENIGMA Sports Injury working group will be driven by the collective effort of the participating members of the working group. Working group chairs provide leadership and support to coordinate efforts and to achieve planned aims. Communication largely relies on regular teleconference calls in addition to face-to-face meetings in alternating locations, typically coinciding with conferences already being attended by the majority of investigators interested in this topic. Overall, we aim to develop a method for open science that allows us to answer important questions that require highly powered studies with large samples of participants.
2. Leverage existing data: It is our mission to leverage existing data by harmonizing data analysis pipelines and subsequently combining data sets from studies on SRC or RHI. In doing so, we will increase the number of data points, thereby improving statistical power when performing secondary analyses. This approach will make it possible to address important questions such as the nature and extent of sex or gender-related differences following SRC and RHI or differences between athlete subgroups such as those participating in sports with different training regimens and access to various levels of medical support. Further, combining many harmonized data sets across studies and cohorts will allow us to explore the integration of multimodal imaging data with clinical outcome measures and fluid biomarkers. This will not only significantly expand our knowledge on the pathophysiology of sport-related brain injury but will also allow identification of biomarkers for prognosis. Ultimately, we aim to inform personalized treatment towards improved outcome after sport-related brain injury. Importantly, members of the consortium decide on their level of participation in each analysis. Such levels of participation include a) mega-analyses (sharing of raw data or output from such data required) b) meta-analyses (no data sharing required), and c) development of methods and analysis tools (no data needed). Further, as part of meetings coinciding with conferences, we plan to offer in-person or hands-on training workshops for data collection, organization, and analysis, and quality assurance techniques. To support participating members with ready-to-use and robust analysis tools to combine existing data sets, ENIGMA offers robust imaging analysis pipelines. These pipelines have been tested across multiple sites by a large number of ENIGMA working groups dedicated to a variety of neurological and psychiatric diseases (Thompson et al. 2020). The respective analysis scripts can be downloaded from the website (http://enigma.ini.usc.edu/protocols/ (ENIGMA n.d.)) and technical support is available.
3. Define key elements of the studies aimed at reducing the acute and chronic effects of sport-related brain injury. The ENIGMA Sports Injury working group is committed to developing and providing recommendations for future prospective data acquisition to enhance the possibilities of data sharing through harmonization of protocols, quality assessment, and post-processing pipelines. As such, short-term goals of the working group are: a) to develop publicly available and user-friendly imaging analysis protocols; b) to determine the sensitivity and specificity of advanced imaging modalities in clinical diagnosis and predicting recovery; and c) to advance methods for harmonization of data analysis and sharing across sites. Our long-term goal is to develop protocols for consistency in data collection where feasible (i.e., new studies beginning data collection) and to further promote open science and mega-analysis of data across sites to answer these important, large-scale questions.

## Summary

Current data from studies of sport-related brain injury is limited by small sample sizes and homogenous cohorts that is being analyzed by a variety of analysis tools. The ENIGMA Sports Injury working group aims to provide a platform for data sharing and collaborative data analysis thereby leveraging existing data and expertise. By harmonizing data from a large number of sources from around the globe, we will collectively work towards reproducibility of previously published results and towards addressing important research questions with regard to diagnosis, prognosis, and recovery of sport-related brain injury. Moreover, the ENIGMA Sports Injury working group is committed to providing recommendations for future prospective data collection to enhance data quality and scientific rigor. The ENIGMA Sport Injury working group invites scientists from around the globe to join this important effort.
